# Perception of Current Educational Environment, Clinical Competency, and Depression among Malaysian Medical Students in Clinical Clerkship: A Cross-Sectional Study

**DOI:** 10.3390/ijerph192316274

**Published:** 2022-12-05

**Authors:** Rosnaini Sudi, Wai Leng Chang, Nur Hidayah Arshad, Syasya Nabilah Zainal Abidin, Ulyssies Suderman, Luke Sy-Cherng Woon

**Affiliations:** Department of Psychiatry, Faculty of Medicine, The National University of Malaysia, Kuala Lumpur 56000, Malaysia

**Keywords:** clinical competency, COVID-19, depression, education environment, medical students

## Abstract

The COVID-19 pandemic has altered the educational environment of medical students in clinical clerkship, with potential impacts on clinical competency and reported increased prevalence of depression. This study aimed to determine the relationship between the perception of the educational environment, self-perceived clinical competency, and depression among them. Subjects (*N* = 196) at the National University of Malaysia participated through convenience sampling in an online survey including sociodemographic data, COVID-19-related stressors, Dundee Ready Education Environment Measure (DREEM), self-perceived clinical competency, and Patient Health Questionnaire (PHQ-9). The cut-off point for depression was a PHQ-9 score ≥ 15. Multiple logistic regression followed bivariate analyses to identify factors for depression. The participants (mean age: 23.2 years, SD ± 0.98 years) were mainly female (71.9%) and Malay (59.2%). The prevalence of depression was 17.4% (95% CI: 12.3–23.4%). Most participants perceived the educational environment positively. In logistic regression, ethnicity (Adjusted OR = 3.1, 95% CI: 1.2–8.1) and DREEM score were significantly associated with depression, whereas self-perceived clinical competency was not. A higher DREEM score indicating a better perception of the educational environment was linked to a lower likelihood of depression (*p* = 0.046). Besides ethnicity, perception of the educational environment emerged as a factor associated with depression. This relationship between the educational environment and mental well-being warrants further exploration.

## 1. Introduction

The COVID-19 pandemic has affected so many lives in different sectors across the globe. One of the sectors severely impacted by the pandemic was medical education in universities around the world [1]. After the initial strict lockdowns that greatly restricted teaching and learning activities in medical schools, most universities have reopened and resumed in-campus learning, although many aspects of medical education have altered compared to the pre-pandemic era. As a result, medical students had to adapt to the unexpected changes in their teaching-learning methods. This could inadvertently affect these medical students’ clinical competency and their mental well-being [2].

In the Faculty of Medicine of the National University of Malaysia (UKM), most lectures, workshops, and tutorials are held online, either through live stream sessions on video conferencing platforms or through asynchronous learning, where all the materials are available at the UKM student online learning portal called UKMFolio. On the other hand, clinical teaching, ward rounds, clinic attachments, and operation theatre observation sessions are conducted face-to-face. However, these sessions are limited as students are required to adhere to the new standards of procedures. This arrangement has likely restricted students’ opportunities to learn in clinical settings as they are required to take turns to have their clinical attachments.

The effects of these educational modifications on medical students’ mental health still require further exploration [3]. Even before the pandemic, medical students have been known to be at high risk for mental health issues [4,5]. A meta-analysis involving 43 countries found that the overall prevalence of depression or depressive symptoms in medical students was 27.2% [6]. The identified risk factors in this population include highly competitive selection processes, intense coursework, pressure for high achievement [4], and sleep deprivation [5,7]. Worryingly, depression among medical students poses an increased risk for suicide and impacts many aspects of professional performance [4,6].

According to a systematic evaluation of the impact of the COVID-19 pandemic on people’s mental well-being, various segments of the population including medical students were affected, leading to the exacerbation of mental health problems such as depression, psychological distress, anxiety, and panic behaviors [8]. Students in a Spanish university experienced anxiety and depressive symptoms when faced with uncertainty in their education, for example, cancellation and postponement of academic activities and the possible poor academic performance that follows [9]. Another study also revealed that students’ adaptation to the new environment of studying medicine at a university with predominant online learning during the COVID-19 pandemic was a great psychological challenge [10]. The sudden shift to online education might have overwhelmed medical students, causing psychological distress [11].

Besides this concern about mental health, there is a further consideration about the impact of the pandemic on medical students’ academic performance, including their clinical competency. For instance, a study conducted at another Malaysian university, Universiti Sains Malaysia, found that many medical students felt worried that they might become less competent clinically due to the pandemic-related changes, including modifications of teaching-learning methods [12]. Similar findings were noted in other countries, with survey data revealing the majority of students reported a reduced feeling of preparedness for beginning their work as a physician because of practicum interruptions [13]. It was noted that the academic performance of medical students as measured by scores or grades in examinations has shown deterioration ever since the conducting of online learning during the COVID-19 pandemic. Meanwhile, resilience among students was associated with better academic performance and lesser mental health issues such as depression, anxiety, and stress [14]. 

There was a significant change in the educational environment of medical students in clinical clerkship after the onset of the COVID-19 pandemic, with uncertain impacts on their clinical competency. At the same time, an increased prevalence of mental health problems especially depression in this population was also observed. It is important to investigate the possible relationship between the current educational environment of medical students in clinical clerkship and their mental health, and the potential role of self-perceived clinical competency in this relationship. The main study objective of this research was to determine the prevalence of depression among medical students in clinical clerkship in UKM. The other objectives were to measure medical students’ perception of the current education environment and their self-perception of their clinical competency. Finally, we investigated the relationship between the perception of the current education environment, clinical competency, and depression among medical students in clinical clerkship.

## 2. Materials and Methods

This was an online survey using a cross-sectional study design. The study population was undergraduate medical students who were in their clinical years (Years 3, 4, and 5) at the Faculty of Medicine, National University of Malaysia (UKM). The inclusion criteria were: (a) Third-year, fourth-year, and fifth-year medical students of the 2021/2022 academic session of UKM; and (b) Providing informed consent to answer the study questionnaire. The exclusion criteria were medical students of other years (first- and second-year medical students of the 2021/2022 academic session) of UKM; postgraduate medical students; respondents who refused to answer the questionnaire; and medical students who were the members of the research team for this study. First- and second-year medical students were not included because they had not yet started their clinical postings.

There were 138 medical students in Year 3, 154 medical students in Year 4, and 124 medical students in Year 5 of study, respectively, at the Faculty of Medicine, UKM at the time of this study, giving a population size of 416. Using the prevalence rate for depressive symptoms of 39% among medical students found in a recent study at another Malaysian public university [15], the sample size for this research study was calculated using the formula for prevalence study (with finite population correction) with the precision (d) = 0.05. The sample size obtained was 195.

The sampling method used in this study was convenience sampling. This was done by distributing the questionnaire in an online survey link to clinical year medical students of UKM through their respective groups in an online messaging platform according to clinical years. This was conducted every week for a duration of two months. To ensure that the number of participants in our study is equivalent for each year, the sample was subdivided into three proportionate blocks. As a result, a total of 64 respondents were needed for Year 3, 72 respondents for Year 4, and 58 respondents for Year 5. Sampling for the respective years was stopped once the required sample size was achieved.

A questionnaire in English was disseminated to clinical year medical students, which consisted of five sections (Sections A–E). Section A was the personal demographic data question questionnaire. This section contained questions regarding personal demographic data, such as gender, age, and year of study, as well as general questions about their financial status, history of medical problems, and history of psychological illness. Section B was a short questionnaire on possible COVID-19-related stressors, such as personal experiences with COVID-19 and its impact on oneself and close friends/relatives. 

Section C was the Dundee Ready Education Environment Measure (DREEM) questionnaire [16]. We used DREEM as a tool to evaluate clinical students’ perception of their current educational environment at their medical school. It is a 50-statement closed-question questionnaire that falls into five subscales: students’ perception of learning, students’ perception of teachers, students’ academic self-perceptions, students’ perception of atmosphere, and students’ social self-perceptions. The statements are scored using a 5-point Likert scale ranging from strongly agree (4) to strongly disagree (0). However, 9 negative statements are scored in a reversed manner. The scores for the items that make up each of the five subscales are added together, and the mean of this sum is used to calculate the subscale summary scores. To obtain the overall DREEM score, the subscale summary scores are summed. The total scores of 0 to 50, 51 to 100, 101 to 150, and 151 to 200 represent perceptions of the current educational environment as “very poor”, “plenty of problems”, “more positive than negative” and “excellent”, respectively. The DREEM instrument has demonstrated high reliability with excellent overall internal consistency (Cronbach’s alpha: 0.92–0.95) and high discriminant and concurrent validities [17,18,19]. In our study, the overall Cronbach’s alpha was 0.94, indicating very good internal consistency. Permission to use the questionnaire had been obtained from its author.

Section D was the Self-Perceived Clinical Competency questionnaire. The questionnaire was on the self-perceived clinical competency of medical students in clinical clerkship. This self-assessment questionnaire consists of 15 questions and was first used by Woolliscroft et al., in a study among 137 third-year medical students from the University of Michigan Hospital who were in internal medicine clerkship to assess themselves on their performances of a variety of clinical skills, knowledge used in a clinical setting, and discharge of their patient care responsibilities [20]. There are 10 self-assessment areas which include medical history interview, physical examination, initial patient write-ups, daily patient progress, oral presentations, application of knowledge, problem list, assessment and plan, self-education, professional responsibilities, and interpersonal interactions. The student self-ratings ranged from 1 (rarely) to 5 (almost always) and the total score was collected by summing up all 15 questions. We employed this questionnaire as it contains questions relevant to our studies of self-perceived clinical competency. Permission to use the questionnaire was obtained from the author. The original study using this questionnaire did not report its psychometric properties. In this current study, the Self-Perceived Clinical Competency questionnaire displayed excellent internal consistency, with a Cronbach alpha value of 0.91.

Lastly, in Section E, depression were evaluated using the Patient Health Questionnaire (PHQ-9) [21]. In this questionnaire, there are nine questions in total, assessing the main symptoms of depression. The total score is calculated by assigning scores of 0, 1, 2, and 3, to the response categories of “not at all,” “several days,” “more than half the days,” and “nearly every day,” respectively, and summing up scores of all nine items. The PHQ-9 total score ranges from 0 to 27. The total score can be categorized into five depression severity categories which are “none to minimal” (0 to 4), “mild” (5 to 9), “moderate” (10 to 14), “moderately severe” (15 to 19), and “severe depression” (20 to 27). The psychometric properties of the 9-item PHQ-9, including its diagnostic validity, are well established. Using PHQ-9 scores > 10 as the cut-off point was found to have a sensitivity of 88% and a specificity of 88% for major depressive disorder. With a cut-off point of 15, the specificity increased to 95% while the sensitivity was 68%. The scale also demonstrated good internal consistency, with Cronbach’s alpha values ranging between 0.86 and 0.89 [21]. In our study, the PHQ-9 had a Cronbach’s alpha value of 0.89.

The Google Forms platform was employed in collecting data. Prioritizing respondents’ confidentiality, the settings of the online questionnaire were adjusted to bypass the requirement of signing in. The respondents filled up the form anonymously and no email addresses were collected. At the beginning of the survey, respondents were reminded to only answer once to prevent multiple submissions from the same respondent. This questionnaire took about 15 to 20 min to complete. Respondents started by reading the subject information sheet followed by filling up the informed consent form. Respondents could only proceed to the actual questionnaire after completing these two steps. At the end of Section E (PHQ-9), participants were provided instructions to calculate and interpret their total scores for PHQ-9. Respondents with a score of 10 and above, representing at least a moderate level of depression [21], were advised to seek further help through proper channels. For instance, seeking support and advice from their mentors in the Faculty’s Mentor-Mentee Program, or going to the Staff-and-student Health Clinic at the university teaching hospital for assessment and referral to the psychiatric clinic, if necessary. 

The data collected was analyzed using the IBM SPSS Version 26.0 software (IBM Corp., Armonk, NY, USA). Descriptive statistics of all study variables were generated. The PHQ-9 score and the DREEM score were further classified, and percentages of the categories of depressive symptoms and perception of the educational environment were calculated. Categories of depressive symptoms were grouped into a dichotomous variable, with a PHQ-9 score of ≥15 (moderately severe depression) classified as depression. For the prevalence of depression in the study population, the 95% confidence interval was calculated. The normality of the distribution of continuous variables was checked with histograms, Q-Q plots, and the Kolmogorov-Smirnov test. Only the PHQ-9 score was not normally distributed. For categorical variables, the PHQ-9 score distribution was compared across categories using the Mann–Whitney U test or the Kruskal–Wallis test. For the continuous variables (age, DREEM score, and self-perceived clinical competency score), correlations with the PHQ-9 scores were calculated using Pearson’s correlation coefficients. Independent variables with a *p*-value of <0.25 were subsequently entered into a logistic regression model with depression as the dichotomous dependent variable. All tests were two-tailed. The significance level (α) was set at 0.05.

This study was carried out after receiving ethical approval from the Research Ethics Committee of the National University of Malaysia (Reference No: UKM PPI/111/8/JEP-2022-312). Written permission was also obtained from the Secretariat of Undergraduate Studies of the Faculty of Medicine, UKM to contact the students to invite them to join this study. Participation in this study was voluntary. Informed consent was obtained from the participants before they proceeded to answer the questionnaire. All the information collected from the questionnaires was kept confidential.

## 3. Results

### 3.1. Sociodemographic Characteristics and COVID-19-Related Questions

A total of 196 students participated in this study, among which 141 (71.9%) were female and 55 (28.1%) were male (Table 1). The mean age was 23.2 years with a standard deviation of ±0.98 years. Most of the participants were from the three major ethnic groups in Malaysia, the highest being Malay (116; 59.2%), followed by Chinese (37; 18.9%) and Indians (32; 16.3%). Among respondents were fourth-year medical students (75; 38.3%), third-year students (64; 32.7%), and fifth-year students (57; 29.1%). This distribution was proportionate to the total number of students in each clinical year.

There were 35 (17.9%) students who failed at least one posting during their clinical years and nine (4.6%) students who had repeated a year of their study. In terms of financial support for their tuition fees, many of the participants were scholarship-holders (141; 71.9%); 33 of them were on a study loan (16.8%) while 22 of them were supported by their families (11.2%). The distribution of monthly household income among the participants was quite consistent with the national norm, with 84 (42.9%) students from M40 (Middle 40%) group (RM 4851–RM 10,970), 62 (31.6%) students from B40 (Bottom 40%) group (≤RM 4850) and 50 (25.5%) students from T20 (Top 20%) group (≥RM 10,971). The majority of the students lived in hostels provided by the university (179; 91.3%). Small proportions of them rented a house or a room (3; 1.5%) or lived with their own families (14; 7.1). Seventeen (8.7%) participants were diagnosed with a psychiatric disorder in the past. The diagnoses recorded include major depressive disorder, persistent depressive disorder, generalized anxiety disorder, bipolar disorder, post-traumatic stress disorder, and others. 

Regarding COVID-19 related questions, a total of 79 (40.3%) students had been infected by the coronavirus and 114 (58.2%) students had not, while 3 (1.5%) students were not sure about their status. Many students had family members and/or close friends who had tested positive for COVID-19 (*n* = 176; 89.9%). In addition, 112 (57.1%) students had experienced direct interaction with COVID-19-positive patients, 73 (37.2%) of them had no direct interaction and 11 (5.6%) of them were not sure. As many as 105 (53.6%) students were worried about being infected with COVID-19, whereas 83 (42.3%) of them were not worried and a minority of them were not sure (*n* = 8; 4.1%). Just over half of the students (*n* = 99; 50.5%) were not worried about surviving if they contracted COVID-19 infection. Only 70 (35.7%) of the students were worried about surviving and the rest were not sure. Notably, most students (78.1%) believed that the COVID-19 pandemic hindered their acquisition of clinical skills, while 30 (15.3%) of them answered no, and 13 (6.6%) of them were not sure.

### 3.2. Perception of the Educational Environment, Self-Perceived Clinical Competency, and Depression

The following are the mean scores and standard deviations for the DREEM (Mean: 131.2, SD: ±19.8), the Self-Perceived Clinical Competency questionnaire (Mean: 54.4, SD: ±7.3) and the PHQ-9 (Mean: 8.7, SD: ±6.0). Based on the scores of the DREEM, only 5.1% of clinical-year medical students perceived the current educational environment as having plenty of problems, while 79.6% perceived that it was more positive than negative and 15.3% agreed that it was excellent (Table 2). The PHQ-9 questionnaire results demonstrated that out of all participants, 31.6% had no depression, 24.5% had mild depression, 26.5% had moderate depression, 14.3% had moderately severe depression, and 3.1% had severe depression. Using a cut-off score of 15 of the PHQ-9 for clinically significant depressive symptoms, the prevalence of depression among clinical year medical students at the National University of Malaysia was 17.4%, with a 95% confidence interval of 12.3% to 23.4%. 

### 3.3. Bivariate Analyses of PHQ-9 Score

In the bivariate analyses of PHQ-9 score across categorical variables, ethnicity (*p* = 0.004) and history of pre-existing psychiatric disorder (*p* = 0.028) were both statistically significant (Table 3). Other variables which had a *p*-value of < 0.25 were a history of failed clinical postings, a history of repeating a year, a history of close family members or friends who tested positive for COVID-19 and worry about surviving a COVID-19 infection. As for the correlations between the PHQ-9 score and other continuous variables, there was a negative correlation between the PHQ-9 score and the DREEM score as the Pearson correlation coefficient was −0.396 with a significance level of < 0.001 (Table 4). A significant negative correlation was also found between the PHQ-9 score and self-perceived clinical competency score with a Pearson correlation coefficient of −0.209 (*p* = 0.003). However, the correlation between the PHQ-9 score and age was not significant (*p* = 0.406). Additionally, a positive correlation was found between the DREEM score and self-perceived clinical competency score (*p* < 0.001).

### 3.4. Logistic Regression Analysis

All variables with a *p*-value of <0.25 (ethnicity, history of pre-existing psychiatric disorder, history of failed clinical postings, history of repeating a year, history of close family members or friends tested positive for COVID-19, and worry about surviving a COVID-19 infection, DREEM score, and self-perceived clinical competency score) were included in the logistic regression model (Table 5). The regression model was statistically significant (Chi-square = 22.460, *p* = 0.013). The Hosmer and Lemeshow test result indicated a good model fit (*p* = 0.720). In this model, after adjusting for other variables, ethnicity and DREEM score remained significantly associated with depression among clinical year medical students, whereas positive psychiatric history and self-perceived clinical competency were no longer significant (Table 4). Compared with non-Malay students, Malays were about three times more likely to experience depression (Adjusted odds ratio, aOR = 3.1, 95% CI: 1.2–8.1). There was a negative association between the DREEM score and depression, with a higher DREEM score linked to a lower likelihood of depression (*p* = 0.046). 

## 4. Discussion

This study investigated the possible relationship between the current educational environment of medical students in clinical clerkship, self-perceived clinical competency, and depression. The prevalence of clinically significant depression among medical students in clinical years was 17.4%. While self-perceived clinical competency was not significantly linked with depression in the logistic regression model, ethnicity and DREEM score were. Malay students had an increased risk of depression by three times. A lower chance of depression was associated with higher DREEM scores, which indicate a better perception of the educational environment.

A study on the prevalence of depression among UKM medical students in clinical clerkship in the pre-pandemic era demonstrated a much lower prevalence (1.3%) as compared to our study. The low prevalence could be due to factors such as the sampling during the post-exam period, medical students adopting good coping strategies, and the implementation of a mentor-mentee programme as a support system [22]. However, another study conducted during the COVID-19 pandemic in 2020 found a higher depression prevalence rate of 36.0% among UKM clinical undergraduate students from medicine, nursing, and paramedics courses. Students who had better social support were less likely to be depressed. Of note, students of Malay ethnicity were reported to have higher stress scores compared to Chinese ethnicity [23]. This discrepancy could be attributed to the cultural differences between these ethnicities [24]. In 2021, a separate study involving UKM both undergraduate and postgraduate medical students investigated the severity of depression, anxiety and stress symptoms after the movement lockdown was lifted. The prevalence rate of depression in this study was relatively high compared to our study at 36.7%. The identified factors included social isolation, reduced emotional support, and loss of recreational activities as a result of social distancing measures [25].

The prevalence of depression in our study (17.4%) was low compared with the prevalence rate for depressive symptoms of 39% among medical students in a recent study at the International Islamic University Malaysia (IIUM) [15]. Another study at the Faculty of Medicine and Health Sciences, Universiti Putra Malaysia that aimed to determine the prevalence and factors associated with depression, anxiety, and stress among medical students found that 31.1% of the respondents were found to be depressed, mainly within the moderately depressed category [26]. The discrepancy could be explained by several factors such as different study tools used to measure depression in the studies, namely PHQ-9 in our study and the Depression Anxiety Stress Scales (DASS-21) in the other studies, different sample sizes, and sociodemographic background of the respondents. Different perceptions of the educational environment among students of the various medical schools could also be a factor.

Additionally, most of our students perceived the current educational environment positively. Another study similarly discovered that the total DREEM scores after the onset of COVID-19 indicated that students in the medical program perceived their educational environment positively. This could be due to the advantages of online learning and being able to learn and revise using online lecture videos at their preferred pace, space, and time in remote learning. The vast amount of online learning materials available could also contribute to a positive perception of the educational environment [27]. Korean medical students perceived that the online learning environment provided a fair chance of enabling communication with their lecturers. Having good interactions with their professors was seen as important in understanding class materials and uplifting their grades [28]. A study involving Singaporean medical students also demonstrated positive participants’ perception of the educational environment despite multiple changes that were made to their curriculum structure during the COVID-19 pandemic [29]. The limitation to the number of medical students in clinical clerkship in compliance to social distancing measures could actually be an advantage rather than a disadvantage. A smaller group of students in the ward enabled them to establish closer and stronger bonds with the in-patients and clinical team, which enhanced individualized feedback and uplifted their motivation [30]. This could lead to increased confidence in students [31]. Having a good support system may also alleviate stress [32].

Medical students’ educational environment has an impact on students’ education, curriculum satisfaction, program and course learning outcomes, and relevant professional development [33]. A few factors that might lead to a positive environment include the teachers being knowledgeable, encouragement to participate in a teaching session, teacher preparedness, good companions, and students’ confidence. Contrarily, factors such as the teacher’s overemphasis on factual learning, overly teacher-centered teaching, and teachers displaying anger in class might lead to students perceiving the educational environment negatively [34,35]. However, the educational environment is not the only factor to create confident, quality students. Even in an optimal learning environment, there may be significant differences in individuals’ learning gains, as factors such as personal learning styles and motivation may ultimately determine a student’s accomplishments [36].

We want to highlight the main finding of our study, namely the significant negative association between the DREEM score and the occurrence of depression among medical students in clinical clerkship. A study involving Australian dentistry students suggested similar findings [37]. Higher DREEM scores indicating a better perception of the educational environment were associated with less psychological distress [38]. This is in line with a study that found that a good learning environment directly reduced medical students’ psychological stress, whereas a hostile environment during medical school increases the likelihood of psychological suffering [39]. The learning environment has a significant impact on the attitude, aspirations, and sense of well-being of medical students. Educational disappointment and negative perceptions can lead to low self-esteem, social and personal neglect, psychosocial morbidities, addiction, aggressiveness, and suicidal attempts. The etiological factors of depression among medical undergraduates could be atmospheric, social, and academic conditions within the medical institution. As shown in a recent study on medical students in Pakistan, dissatisfaction with the examination schedule and academic burden were the main factors associated with depression [40]. A good perception of the educational setting may help to create a healthy environment for the students, physically and emotionally.

However, a reverse relationship is also possible, where the presence of depression leads to a negative perception of the educational environment. Negative thinking, such as brooding and rumination, is a common associated feature of depression [41]. The cognitive theory of depression postulates that individuals with depression hold negative thoughts about themselves, their experiences in the world, and their future [42]. The cognitive bias in depression can cause preferential recall of negative memories, and there is a possibility of higher attention to negative events and a bias towards negative interpretations [43]. In the context of the present study, it might mean that respondents with preexisting depression had a greater tendency to rate their educational environment unfavorably because of the biased cognitive processing they experienced. Hence, further studies are needed to investigate how depression may affect the perception of the educational environment and vice versa. At any rate, it is important to raise awareness and understanding of a good learning environment to create a less stressful and more manageable learning environment for all students. 

A significant demographic factor associated with depression among medical students in clinical clerkship was found to be ethnicity. In this study, most respondents were Malays followed by Chinese, who made up the bulk of the non-Malay study subjects. In a previous study on Malaysian medical students, Chinese students reported having better mental health than the others, indicating that ethnicity and mental health could be connected [44]. Interestingly, our study further confirmed the relationship between ethnicity and depression as it was suggested that Malay students were more likely to have depression as compared to Chinese students. Chinese ethnicity appears to be protective against depression but the precise mechanisms are unknown. From a cultural perspective, previous research has shown that Chinese people tend to somatize rather than psychologize their problems [45]. This could be one possible explanation for the lower reported rates of depression among medical students of Chinese descent. As for Malay ethnicity, previous researchers have also postulated that cultural factors might contribute to the higher likelihood of mental ill-health in this demographic group [46], but the unique sociocultural causal factors of depression should be further explored. 

We found that there was no significant association between self-perceived clinical competency and depression. It could possibly because self-perceived clinical competency was not a good representation of medical students’ actual clinical competency. For instance, correlation between self-perceived clinical competency and objective competency was lacking in a group of final-year medical students [47]. Self-perception of clinical competency involves self-evaluation. For self-evaluation to be accurate, there must be a self-representation of the actual performance that is consistent with reality. Self-representation is often based on feedback. However, over time, self-representations may grow more resistant to change when students are confronted with feedback that contradicts them. Contrasting and comparing one’s performance with that of role models, such as teachers and senior clinicians, is one of the ways that people might develop appropriate self-assessment skills [20]. Thus, results gained from a self-perceived clinical competency questionnaire may not be as accurate as desired because it could have been influenced by many factors such as self-confidence and role models. 

There were a few limitations to this study. The cross-sectional study design only allowed data collection at one point in time; therefore, it could not determine the causal relationship between the variables being studied. The convenience sampling used in this study could have given rise to sampling bias. To comply with the Ethics Committee’s requirement of preserving anonymity, we did not record the identity of respondents and therefore were unable to entirely rule out the possibility of repeated responses by the same respondents. Only self-perceived clinical competency was measured without an objective measurement of the clinical competency of the medical students, which might limit the validity of the measure of clinical competency. The prevalence of depression found in this study was based on a screening instrument, not a clinical diagnostic tool. Other important psychiatric symptoms, such as anxiety symptoms, were not studied in this survey. As our study population only involved clinical-year undergraduate medical students in the Faculty of Medicine, National University of Malaysia, the results may not be representative of a larger national population. 

Despite the limitations, our study was able to investigate the prevalence of depression among clinical students in a post-pandemic setting, taking note of their current educational environment and their clinical competency perceptions, which, to the best of our knowledge, have not been studied together before. Various sociodemographic, academic, and COVID-19-related variables were included in this survey to control for potential confounders. This study also managed to investigate the students’ perceptions of the current educational environment in the Faculty of Medicine of the National University of Malaysia, which were mostly positive. This finding may contribute to the development and refinement of the medical education program at the university.

## 5. Conclusions

Medical students have to adapt to the abrupt changes in their teaching-and-learning method in the post-pandemic era, which raises the question of whether these changes would impact students’ clinical competency and trigger mental health issues. Our survey suggested that although the majority of UKM medical students perceived their educational environment positively, a poorer perception of the educational environment was associated with poorer mental health, irrespective of the levels of their perceived clinical competency. An educational environment perceived as conducive might be related not only to better performance but also to a lesser likelihood of depression. Future longitudinal studies may help elucidate the causal relationship between educational environment and depression among medical students in clinical clerkship. Strategies for the improvement of the undergraduate medical educational environment may potentially enhance their mental well-being.

## Figures and Tables

**Table 1:** Sociodemographic information of respondents and COVID-19-related questions.

Variable	*n* (%), *N* = 196
Gender	
Female	141 (71.9)
Male	55 (28.1)
Ethnicity	
Malay	116 (59.2)
Chinese	37 (18.9)
Indian	32 (16.3)
Others	11 (5.6)
Year of study	
Year 3	64 (32.7)
Year 4	75 (38.3)
Year 5	57 (29.1)
Have failed at least one posting during clinical years	
Yes	35 (17.9)
No	161 (82.1)
Have repeated a year in medical school	
Yes	9 (4.6)
No	187 (95.4)
Source of financial support	
Scholarship	141 (71.9)
Study loan	33 (16.8)
Family	22 (11.2)
Monthly household income	
≤RM 4850 (B40)	62 (31.6)
RM 4851–RM 10,970 (M40)	84 (42.9)
≥RM 10,971 (T20)	50 (25.5)
Place of residence	
Hostel	179 (91.3)
Rented house/room	3 (1.5)
Family home	14 (7.1)
Diagnosed with a psychiatric disorder in the past	
Yes	17 (8.7)
No	179 (91.3)
Tested positive for COVID-19	
Yes	79 (40.3)
No	114 (58.2)
Not sure	73 (1.5)
Any close family members and/or friends tested positive for COVID-19	
Yes	176 (89.8)
No	19 (9.7)
Not sure	1 (0.5)
Had any direct interactions with COVID-19-positive patients	
Yes	112 (57.1)
No	73 (37.2)
Not sure	11 (5.6)
Worried that he/she has been infected with COVID-19	
Yes	105 (53.6)
No	83 (42.3)
Not sure	8 (4.1)
Worried about surviving if he/she got COVID-19 infection	
Yes	70 (35.7)
No	99 (50.5)
Not sure	27 (13.8)
Thinks that the COVID-19 pandemic hinders his/her acquisition of clinical skills	
Yes	153 (78.1)
No	30 (15.3)
Not sure	13 (6.6)

B40, Bottom 40% income group; M40, Middle 40% income group; T20, Top 20% income group.

**Table 2:** DREEM and PHQ-9 categories.

Variable	*n* (%), *N* = 196
DREEM category	
Plenty of problems	10 (5.1)
More positive than negative	156 (79.6)
Excellent	30 (15.3)
PHQ-9 category	
No depression	62 (31.6)
Mild depression	48 (24.5)
Moderate depression	52 (26.5)
Moderately severe depression	28 (14.3)
Severe depression	6 (3.1)

DREEM, Dundee Ready Education Environment Measure; PHQ-9, Patient Health Questionnaire 9.

**Table 3:** Bivariate analyses of PHQ-9 score across categorical variables.

Variable	Depression *n* (%)	No Depression *n* (%)	*p*-Value
Gender			0.876 ^a^
Male	10 (18.2)	45 (81.8)	
Female	24 (17.0)	117 (83.0)	
Ethnicity			0.004 ^b,^*
Malay	27 (23.3)	89 (76.7)	
Chinese	2 (5.4)	35 (94.6)	
Indian	4 (12.5)	28 (87.5)	
Others	1 (9.1)	10 (90.9)	
Year of study			0.647 ^b^
Year 3	10 (15.6)	54 (84.4)	
Year 4	14 (18.7)	61 (81.3)	
Year 5	10 (17.5)	47 (82.5)	
Failed posting			0.085 ^a^
Yes	9 (25.7)	26 (74.3)	
No	25 (15.5)	136 (84.5)	
Repeat year			0.213 ^a^
Yes	4 (44.4)	5 (55.6)	
No	30 (16.0)	157 (84.0)	
Source of financial support			0.505 ^b^
Family	4 (18.2)	18 (81.8)	
Study loan	7 (21.2)	26 (78.8)	
Scholarship	23 (16.5)	116 (83.5)	
Monthly household income			0.605 ^b^
B40	14 (22.6)	48 (77.4)	
M40	10 (11.9)	74 (88.1)	
T20	10 (20.0)	40 (80.0)	
Place of residence			0.908 ^a^
Hostel	32 (17.9)	147 (82.1)	
Rented house/room	0 (0.0)	3 (100.0)	
Family home	2 (14.3)	12 (85.7)	
Diagnosed with a psychiatric disorder			0.028 ^a,^*
Yes	6 (35.5)	11 (64.7)	
No	28 (15.6)	151 (84.4)	
Tested positive for COVID-19			0.622 ^b^
Yes	14 (17.7)	65 (82.3)	
No	20 (17.5)	94 (82.5)	
Not sure	0 (0.0)	3 (100.0)	
Any close family and/or close friends tested positive for COVID-19			0.164 ^b^
Yes	31 (17.6)	145 (82.4)	
No	3 (15.8)	16 (84.2)	
Not sure	0 (0.0)	1 (100.0)	
Had any direct interactions with COVID-19-positive patients			0.835 ^b^
Yes	19 (17.0)	93 (83.0)	
No	13 (17.8)	60 (82.2)	
Not sure	2 (18.2)	9 (81.8)	
Worried that he/she has been infected with COVID-19			0.425 ^b^
Yes	20 (19.0)	85 (81.0)	
No	14 (16.9)	69 (83.1)	
Not sure	0 (0.0)	8 (100.0)	
Worried about surviving if he/she contracted COVID-19 infection			0.178 ^b^
Yes	11 (15.7)	59 (84.3)	
No	17 (17.2)	82 (82.8)	
Not sure	6 (22.2)	21 (77.8)	
Thinks that the COVID-19 pandemic hinders his/her acquisition of clinical skills			0.301 ^b^
Yes	29 (19.0)	124 (81.0)	
No	3 (10.0)	27 (90.0)	
Not sure	2 (15.4)	11 (84.6)	

B40, Bottom 40% income group; M40, Middle 40% income group; T20, PHQ-9, Patient Health Questionnaire 9; Top 20% income group; ^a^ Mann–Whitney U test; ^b^ Kruskal–Wallis Test; * Statistically significant.

**Table 4:** Correlations between PHQ-9 score and other continuous variables.

Variable	Pearson’s r	*p*-Value
Age	0.060	0.406
DREEM score	−0.396	<0.001 *
Self-perceived clinical competency score	−0.209	0.003 *

DREEM, Dundee Ready Education Environment Measure; PHQ-9, Patient Health Questionnaire 9; * Statistically significant.

**Table 5:** Logistic regression analysis for factors associated with depression.

Variable	Adjusted OR	95% CI		*p*-Value
		Lower	Upper	
Ethnicity				
Malay	1.0			
Non-Malay	3.1	1.2	8.1	0.020 *
Have you ever failed any postings during your clinical years?				
No	1.0			
Yes	1.3	0.5	3.4	0.612
Have you ever repeated a year in medical school?				
No	1.0			
Yes	4.9	1.0	24.4	0.054
Have you been diagnosed with a psychiatric disorder in the past?				
No	1.0			
Yes	2.1	0.7	6.8	0.215
Has any of your close family and/or close friends tested positive for COVID-19?				
No	1.0			
Yes	1.4	0.3	5.6	0.677
Unsure	0.0	0.0		1.000
Do you worry about surviving if you get a COVID-19 infection?				
No	1.0			
Yes	0.8	0.3	2.0	0.665
Unsure	1.0	0.3	3.5	0.967
DREEM score	1.0	1.0	1.1	0.046 *
Self-perceived clinical competency score	1.0	0.9	1.1	0.497

χ^2^ = 22.460, df = 10, *p* < 0.013; Nagelkerke R^2^ = 0.180; * Statistically significant.

## Data Availability

The data presented in this study are available on request from the corresponding author. The data are not publicly available due to privacy and confidentiality policy at the study site.
